# Intelligent Multirobot Navigation and Arrival-Time Control Using a Scalable PSO-Optimized Hierarchical Controller

**DOI:** 10.3389/frai.2020.00050

**Published:** 2020-08-07

**Authors:** Yu-Cheng Chang, Anna Dostovalova, Chin-Teng Lin, Jijoong Kim

**Affiliations:** ^1^Computational Intelligence and Brain Computer Interface (CIBCI) Lab, Centre for Artificial Intelligence (CAI), University of Technology, Sydney, NSW, Australia; ^2^Defence Science & Technology Group, Adelaide, SA, Australia

**Keywords:** hierarchical fuzzy system, fuzzy logic control, multi-agent control, navigation, arrival-time control

## Abstract

We present a hierarchical fuzzy logic system for precision coordination of multiple mobile agents such that they achieve simultaneous arrival at their destination positions in a cluttered urban environment. We assume that each agent is equipped with a 2D scanning Lidar to make movement decisions based on local distance and bearing information. Two solution approaches are considered and compared. Both of them are structured around a hierarchical arrangement of control modules to enable synchronization of the agents' arrival times while avoiding collision with obstacles. The proposed control module controls both moving speeds and directions of the robots to achieve the simultaneous target-reaching task. The control system consists of two levels: the lower-level individual navigation control for obstacle avoidance and the higher-level coordination control to ensure the same time of arrival for all robots at their target. The first approach is based on cascading fuzzy logic controllers, and the second approach considers the use of a Long Short-Term Memory recurrent neural network module alongside fuzzy logic controllers. The parameters of all the controllers are optimized using the particle swarm optimization algorithm. To increase the scalability of the proposed control modules, an interpolation method is introduced to determine the velocity scaling factors and the searching directions of the robots. A physics-based simulator, Webots, is used as a training and testing environment for the two learning models to facilitate the deployment of codes to hardware, which will be conducted in the next phase of our research.

## Introduction

Mobile robot control has been widely used in automated navigation system. The aim of the automated navigation is to guide the robot or vehicle moving between obstacles to reach the target from the start point with collision-free performance (Kashyap and Pandey, 2018; Patle et al., 2019). During the navigation control, observations are made by the sensors and actuators equipped on a real robot or vehicle; the input signal is noisy and uncertain. Therefore, fuzzy logic system (FLS) (Zhu and Yang, 2007; Juang and Chang, 2011; Pothal and Parhi, 2015; Lai et al., 2016; Din et al., 2018; Jhang et al., 2019; Mohanta and Keshari, 2019; Pradhan et al., 2019) has been used in an automated navigation takes in order to enhance the robot control quality. Fuzzy logic systems (FLSs) provides a robust solution with anti-noise ability to defeat the uncertainty. However, the performance of fuzzy logic system depends on the design of membership function and efficient rules, which often takes a considerable amount of time to analyze the experimental input and output data. Machine learning technology, therefore, has been used for fuzzy system design. Zhu and Yang (2007) and Pothal and Parhi (2015), respectively, exploit supervised learning to train neuro-fuzzy model for single and multiple robots to perform navigation task. The precise input–output training data should be collected in advance for supervised learning. To reduce the training effort, evolutionary algorithms have been used to design FLS. Two popular optimization algorithms are genetic algorithms (GAs) (Chia-Feng, 2005; Mansoori et al., 2008; Nantogma et al., 2019; Pradhan et al., 2019) and particle swarm optimization (PSO) (Juang and Lo, 2008; Juang and Chang, 2011; Ding et al., 2019). These two methods can be easily applied to the design of FLS since it can be formulated as an optimization problem by defining a metric for solution performance evaluation.

Apart from single-agent navigation, the last few years have seen an increase in research topic (Pothal and Parhi, 2015; Misra et al., 2018; Babel, 2019; Chandrasekhar Rao and Kabat, 2019; Yao and Qi, 2019) for multirobot coordination across multiple disciplines. The multirobot coordination entails time synchronization among individuals to accomplish a given task. This study considers time-arrival control during the multiple robots performing navigation task; the robots not only need to move toward their targets with collision-free motion in a timely manner. Yao and Qi (2019) propose a novel dynamical model to adjust the path length and voyage speed of each autonomous underwater vehicle (AUV) to achieve the simultaneous arrival at destination between multi-AUVs. Misra et al. (2018) combine cooperative localization technique with proportional navigation (PN) guidance law to manipulate multiple unmanned vehicles to simultaneously reach a moving target in GPS-denied environment. Babel (Lin and Lee, 1996; Chia-Feng and Chin-Teng, 1998) develops a multi-agent path planning algorithm considering shortest paths between all pairs of air vehicles and targets, target allocation, and concatenating feasible and suitable short path, which guarantees that all UAVs arrive at the targets in a timely manner and without the risk of mutual collision.

This paper developed a fuzzy-based control system that has two levels: the lower-level individual navigation control for obstacle avoidance and the higher-level coordination to ensure the same time of arrival for all robots at their destination points. An FLS methodology (Lin and Lee, 1996; Chia-Feng and Chin-Teng, 1998; Juang and Chang, 2011), combining the Takagi-Sugeno-Kang (TSK) type of a fuzzy inference system with a derivative-free global optimization technique, is used to design the fuzzy “IF–THEN” rules and tune the parameters of membership functions. The controllers are trained in a cascading manner. In the first phase of training, we employ the particle swarm optimization (PSO) algorithm (Shi and Eberhart, 1998) to optimize the fuzzy rules that comprise individual navigation control. The role of this controller is to generate the motion direction command that steers the robot away from obstacles but toward the target location based on the robot's sensory inputs (each robot is equipped with a laser ranger) and information about target location. In the second phase of training, the same technique is used for multiple robots to learn how to coordinate with each other to reach their targets at the same time. The coordination controller controls both moving speeds and moving direction of each robot to achieve the simultaneous target reaching task. We developed a fuzzy-logic-based coordinator and a recurrent-based coordinator. The fuzzy-logic-based coordinator is implemented by a PSO-optimized FLS. The recurrent-based coordinator includes a PSO-trained long short-term memory (LSTM) block (Greff et al., 2017). Webots software (Webots: Robot simulator) has been used as a physics-based robot simulation environment for the training and testing of the proposed solutions.

## The Proposed Models

**Figure 1:**
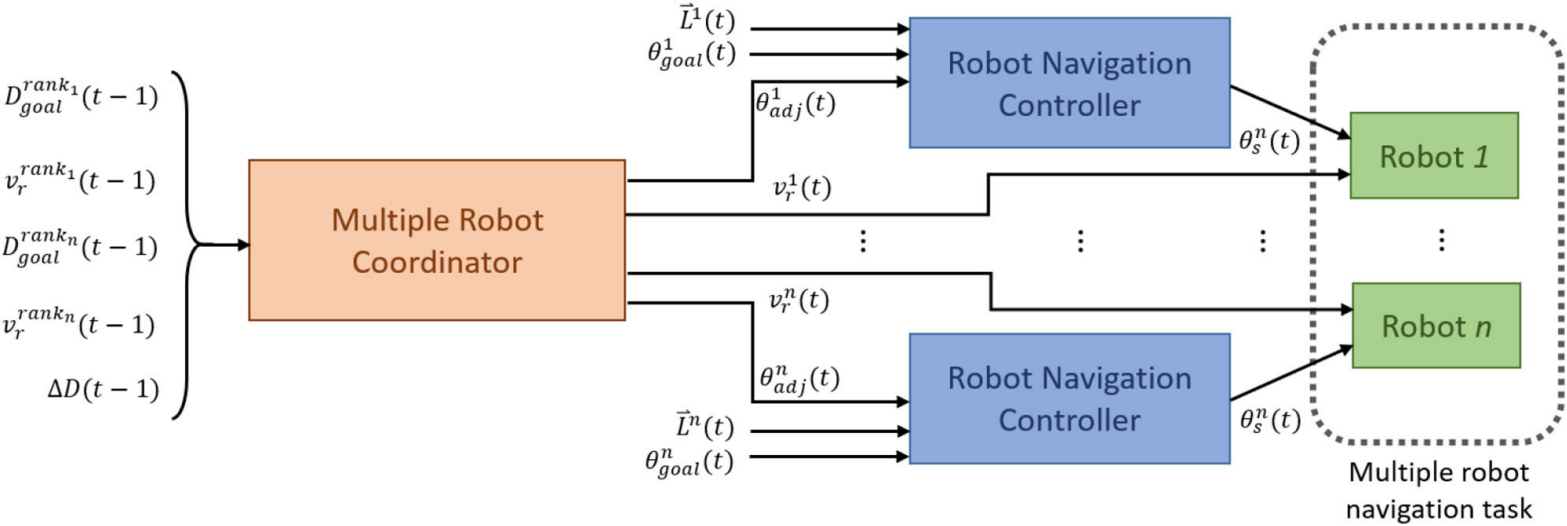
The block diagram of the control configuration for multirobot navigation and arrival-time control.

This section describes the proposed models for multirobot navigation and arrival-time control. Figure 1 shows the configuration. There are two hierarchical levels of control module. The lower-level controllers are the robot navigation controllers (RNCs). They enable each robot to perform collision-free navigation. The higher-level controller is the multiple robot coordinator (MRC), which coordinates the robots' speed and direction so that they reach their targets at the same time.

**Figure 2:**
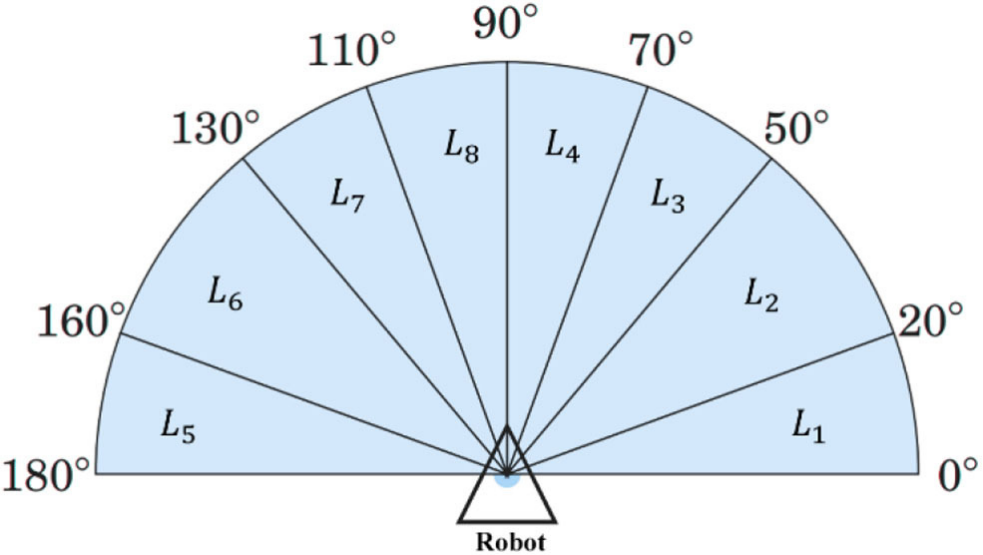
Scanning area of the 2D Lidar in the simulation setting.

In the proposed model, each RNC controls a robot, and all RNCs share an identical structure and a set of parameters. The RNC receives the adjustment angle, i.e., θ_*adj*_(*t*), from the output of MRC, the distances between the robot and nearby obstacles, i.e., $\overset{⇀}{L}$(*t*) from a 2D Lidar and the direction angle to the target from the robot, i.e., θ_*goal*_(*t*). Specifically, a 2D Lidar rangefinder on the front of the robot scans from 0 to 180° and outputs $\overset{⇀}{L}$(*t*) = (*L*_1_, …, *L*_8_), which are the minimum distances to any obstacle in each of eight sectors (Figure 2). The output of an RNC is the motion direction of the robot. To achieve collision-free navigation, a fuzzy logic controller (FLC) was added to the RNC. The robot avoids obstacles using boundary-following (BF) behavior. A behavior supervisor (BS) decides what the robot should do at each control time step.

The MRC is a centralized controller to determine the speed and direction of each robot for the next control time step with five inputs: $D_{goal}^{rank_{1}}\left(t-1\right)$, $D_{goal}^{rank_{n}}\left(t-1\right)$, $v_{r}^{rank_{1}}\left(t-1\right)$, $v_{r}^{rank_{n}}\left(t-1\right)$, and Δ*D*(*t*−1). For each control loop, the robots are ranked in ascending distance from the target. Accordingly, *robot*_*rank*_1__ is the closest robot to the target, and *robot*_*rank*_*n*__ is the robot farthest away. $D_{goal}^{rank_{1}}$ and $D_{goal}^{rank_{n}}$ are, respectively, the distances from *robot*_*rank*_1__ and *robot*_*rank*_*n*__to the target. $v_{r}^{rank_{1}}$ and $v_{r}^{rank_{n}}$ are, respectively, the speeds of *robot*_*rank*_1__ and *robot*_*rank*_*n*__. Finally, $\Delta D\left(t-1\right)=D_{goal}^{rank_{n}}\left(t-1\right)-\;D_{goal}^{rank_{1}}\left(t-1\right)$. The outputs of MRC to the RNCs are the speed *v*_*r*_(*t*) and heading angle θ_*adj*_(*t*) for robot, *rank*_1_, …, *rank*_*n*_. We developed two types of MRC, one with a fuzzy-logic-based model and the other with a recurrent-based model. The fuzzy-logic-based MRC is implemented by an FLC, while the recurrent-based model uses long short-term memory (LSTM). Details of the proposed models are introduced in the following sections.

### Fuzzy-Logic-Based Multiple Robot Coordinator

The proposed fuzzy-logic-based MRC is responsible for speed regulation and heading angle adjustment. The robot speed is changed at each control time step. If a robot is much closer to the target than the other robots, heading angle adjustment is made so that it moves away from the target; otherwise it would need to stop and wait for the others.

#### Speed Regulation

For each control loop, *n* robots are ranked based on their distance to the target in ascending order. An FLC called *FLC*_*SR*_ is used for robot speed regulation, which directly controls *robot*_*rank*_1__ and *robot*_*rank*_*n*__. The outputs of *FLC*_*SR*_ are speed factors α_1_ and α_*n*_ for these two robots, which are used to increase or decrease their speeds. For the remaining robots (*robot*_*rank*_2__, …, *robot*_*rank*_(*n*−1)__), the speed scale factors α_2_, …, α_(*n*−1)_ are generated by the following interpolation process:

(1) $$\begin{array}{l} \alpha_{i}=\frac{i-1}{n-1}\cdot \left(\alpha_{n}-\alpha_{1}\right)+\alpha_{1}, \end{array}$$

where *i* = 2, …, *n* − 1, and α_*i*_ ∈ [0.5, 1.5]. The speeds have upper and lower bounds, which define a safe operating region.

The robots are not allowed to stop. The speeds for the robots at control time step *t* are given by

(2) $$\begin{array}{l} v_{r}^{i}\left(t\right)\;=\alpha_{i}v_{r}^{i}\left(t-1\right),\;i=1,\ldots ,n. \end{array}$$

*FLC*_*SR*_ uses zero-order Takagi–Sugeno–Kang (TSK) fuzzy IF–THEN rules with the form

(3) $$\begin{array}{l} R_{i}^{SR}:\text{If }x_{1}\text{ is }A_{i1}\text{ And }\ldots \text{ And }x_{5}\text{ is }A_{i5}\text{ Then }y\text{ is }{\overset{⇀}{a}}_{i} \end{array}$$

where *x*_1_, …, *x*_5_ correspond to the input variables; $D_{goal}^{rank_{1}}\left(t-1\right)$, $D_{goal}^{rank_{n}}\left(t-1\right)$, $v_{r}^{rank_{1}}\left(t-1\right)$, $v_{r}^{rank_{n}}\left(t-1\right)$, and *D*(*t*−1); *A*_*i*1_,…, *A*_*i*5_ are fuzzy sets; and ${\overset{⇀}{a}}_{i}=\left(a_{i1},a_{i2}\right)$ is a real vector. Here we use a Gaussian membership function. Thus, *A*_*ij*_ is given by

(4) $$\begin{array}{l} \mu_{ij}\left(x_{i}\right)=exp\left\{-{\left(\frac{x_{i}-m_{ij}}{\sigma_{ij}}\right)}^{2}\right\} \end{array}$$

where *m*_*ij*_ and σ_*ij*_ represent the center and the width of the fuzzy set *A*_*ij*_, respectively. The firing strength of rule $R_{i}^{SR}$ is obtained by implementing the following algebraic product:

(5) $$\begin{array}{l} \Phi_{i}=\overset{M}{\underset{j=1}{\prod }}\mu_{ij}\left(x_{i}\right), \end{array}$$

where *M* is the dimension of the input variable, e.g., *M* = 5.

Suppose that *FLC*_*SR*_ has *r* rules. An output $\overset{⇀}{y}=\left(y_{1},y_{2}\right)=\left(\alpha_{1},\alpha_{n}\right)$ can be obtained using the weighted average defuzzification method:

(6) $$\begin{array}{l} \overset{⇀}{y}=\frac{\sum_{i=1}^{r\;}\Phi_{i}{\overset{⇀}{a}}_{i}}{\sum_{i=1}^{r}\Phi_{i}}. \end{array}$$

#### Heading Angle Adjustment

**Figure 3:**
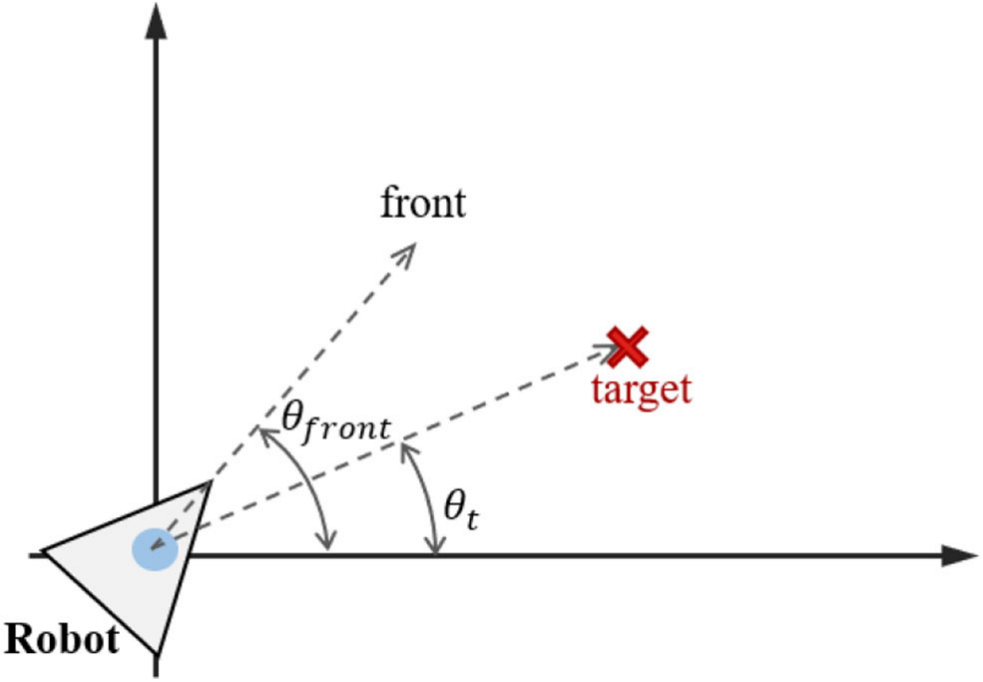
Target angle and robot orientation angle.

To make the length of the robot paths roughly equal, the MRC adjusts each robot's heading angle as part of its arrival-time control. An adjusted heading angle for robot *i* at control time step *t* is calculated by

(7) $$\begin{array}{l} \theta_{adj}^{i}\left(t\right)=\theta_{goal}^{i}\left(t\right)+{\beta_{i}\cdot \theta }_{\max\text{\_}adj}, \end{array}$$

where β_*i*_ is a scale factor that determines the strength of heading angle adjustment, $\theta_{goal}^{i}$ is the search angle for robot *i*, and θ_max_*adj*_ is the maximum angle in changing the direction of robot *i*, e.g., θ_max_*adj*_ = 90. $\theta_{goal}^{i}=\theta_{t}-\theta_{front}^{i}$ is the deviation between the target angle θ_*t*_ and the robot orientation angle $\theta_{front}^{i}$, as shown in Figure 3. β_*i*_ is calculated from $D_{goal}^{rank_{n}}$, $D_{goal}^{i}$ and α_*i*_, as follows:

(8) $$\begin{array}{l} \beta_{i}=\sqrt{\frac{1}{\alpha_{i}}\left(1-\frac{D_{goal}^{i}\left(t-1\right)}{D_{goal}^{rank_{n}}\left(t-1\right)}\right)} \end{array}$$

As β_*i*_ increases, robot *i* will be guided away from the target; on the other hand, as β_*i*_ decreases, robot *i* will be guided toward the target. This adjustment keeps changing the search behavior of each robot, except for the furthest *robot*_*rank*_*n*__, until either $(D_{goal}^{rank_{n}}-D_{goal}^{rank_{1}})<0.1\;m$ or each robot is within 10 *m* of the target. Algorithm 1 is an overview of the fuzzy-logic-based MRC for the multirobot navigation and arrival-time control.

**Algorithm 1:** Pseudocode for the multirobot navigation and arrival-time control.

*Initialize the robots*
**for all** robots *i* **do**
Get initial position
Set the position of the target
Set initial moving speed to 0.5 m/s
**end for**
*Main control loop*
**while** stop conditions have not been met **do**
**for all** robots *i* **do**
$L_{1}^{i},\ldots ,L_{8}^{i}\leftarrow$ Lidar output
Get distance to the target $D_{goal}^{i}$
**end for**
Rank all robots according to $D_{goal}^{i}$
$\Delta D\leftarrow D_{goal}^{rank_{n}}-\;D_{goal}^{rank_{1}}$
α_1_, α_*n*_ ← *FLC*_*SR*_(*v*_*r*1_, *v*_*r*2_, *D*_*g*1_, *D*_*gn*_, Δ*D*)
**for** robot *i* **from** *robot*_*rank*_2__ to *robot*_*rank*_(*n*−1)__
α_*i*_ ← equation (1)
**end for**
**for all** robots *i* **do**
$v_{r}^{i}\;\leftarrow \text{equation }\left(2\right)$
**if** (robot *i* is performing TS behavior **and**
(Δ*D* > 0.1 *m* **or** $D_{goal}^{rank_{n}}>10\;m$))
β_*i*_ ← equation (8)
$\theta_{adj}^{i}\leftarrow \text{equation }\left(7\right)$
**else**
$\theta_{adj}^{i}\leftarrow \theta_{goal}^{i}$
**end if**
**end for**
**for all** robots *i* **do**
Steering angle $\theta_{r}^{i}\leftarrow RBC\left(L_{1}^{i},\ldots ,L_{8}^{i},D_{goal}^{i},\;\theta_{adj}^{i}\right)$
**end for**
**end while**

### Recurrent-Based Multiple Robot Coordinator

**Figure 4:**
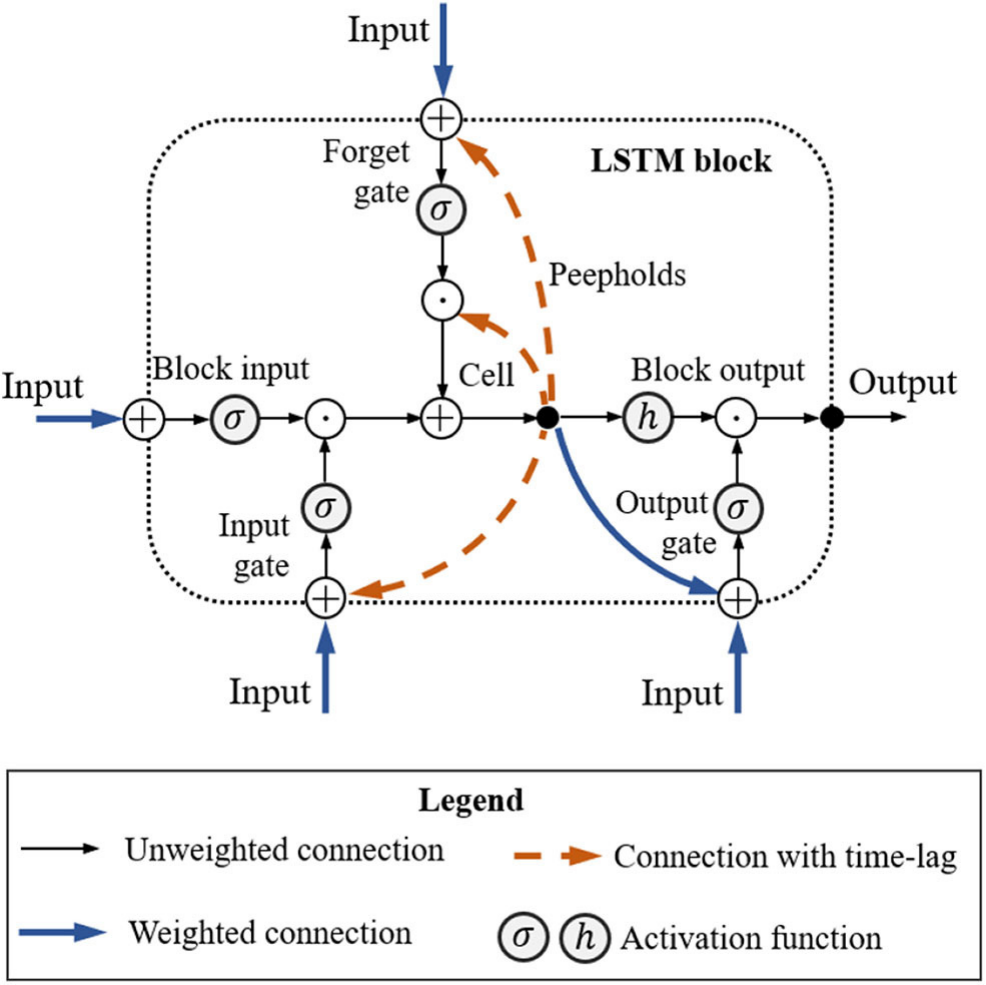
Long short-term memory block.

In addition to fuzzy-logic-based MRC, we also consider an LSTM-based model because these perform well for problems involving sequential data with long time dependencies. Its memory mechanism allows the use of historical data, which could be useful for optimizing trajectory-related problems. The vanilla version of LSTM is used because it is simple to implement, and its performance is close to that of other variants. Figure 4 shows the architecture of the LSTM block. In the recurrent-based MRC configuration, we use two LSTM blocks, with input (*W*_*z*_, *W*_*i*_, *W*_*f*_, *W*_*o*_), recurrent (*R*_*z*_, *R*_*i*_, *R*_*f*_, *R*_*o*_), peephole (*p*_*i*_, *p*_*f*_, *p*_*o*_), and bias (*b*_*z*_, *b*_*i*_, *b*_*i*_, *b*_*o*_) weights. The input/output interface of the LSTM controller matches that of the fuzzy-logic-based MRC. Given input $x^{k}=\left(D_{goal}^{rank_{1}}\left(t-1\right),\;D_{goal}^{rank_{n}}\left(t-1\right)\;v_{r}^{rank_{1}}\left(t-1\right),\;v_{r}^{rank_{n}}\left(t-1\right),\;\Delta D\left(t-1\right)\right)$, then the LSTM block forward pass is

(9) $$\begin{array}{lll} \text{Block input}:\;z^{k} & = & h\left(W_{z}{\text{x}}^{k}+R_{z}{\text{y}}^{k-1}+b_{z}\right), \end{array}$$

(10) $$\begin{array}{lll} \text{Input gate}:\;i^{k} & = & \sigma \left(W_{i}{\text{x}}^{k}+R_{i}{\text{y}}^{k-1}+p_{i}⊙c^{k-1}+b_{i}\right), \end{array}$$

(11) $$\begin{array}{lll} \text{Forget gate}:\;f^{k} & = & \sigma \left(W_{f}{\text{x}}^{k}+R_{f}{\text{y}}^{k-1}+p_{f}⊙c^{k-1}+b_{i}\right), \end{array}$$

(12) $$\begin{array}{lll} \text{Cell}:\;c^{k} & = & z^{k}⊙i^{k}+c^{k-1}⊙f^{k}, \end{array}$$

(13) $$\begin{array}{lll} \text{Output gate}:\;o^{k} & = & \sigma \left(W_{o}{\text{x}}^{k}+R_{o}{\text{y}}^{k-1}+p_{o}⊙c^{k}+b_{o}\right), \end{array}$$

(14) $$\begin{array}{lll} \text{Block output}:\;y^{k} & = & h\left(c^{k}\right)⊙o^{k}, \end{array}$$

where σ is the logistic sigmoid function used for gate activation, and *h* is the hyperbolic tangent function for the block input/output activation. During the training process, all bias weights are set to 0.5.

### Robot Behavior Controller

The robot behavior controller (RBC) controls a robot to avoid obstacles by performing left BF behavior or right BF behavior. It consists of a behavior supervisor (BS) to determine behavior according to its current position, target position, and real-time outputs from the 2D Lidar sensor.

#### Behavior Supervisor

**Figure 5:**
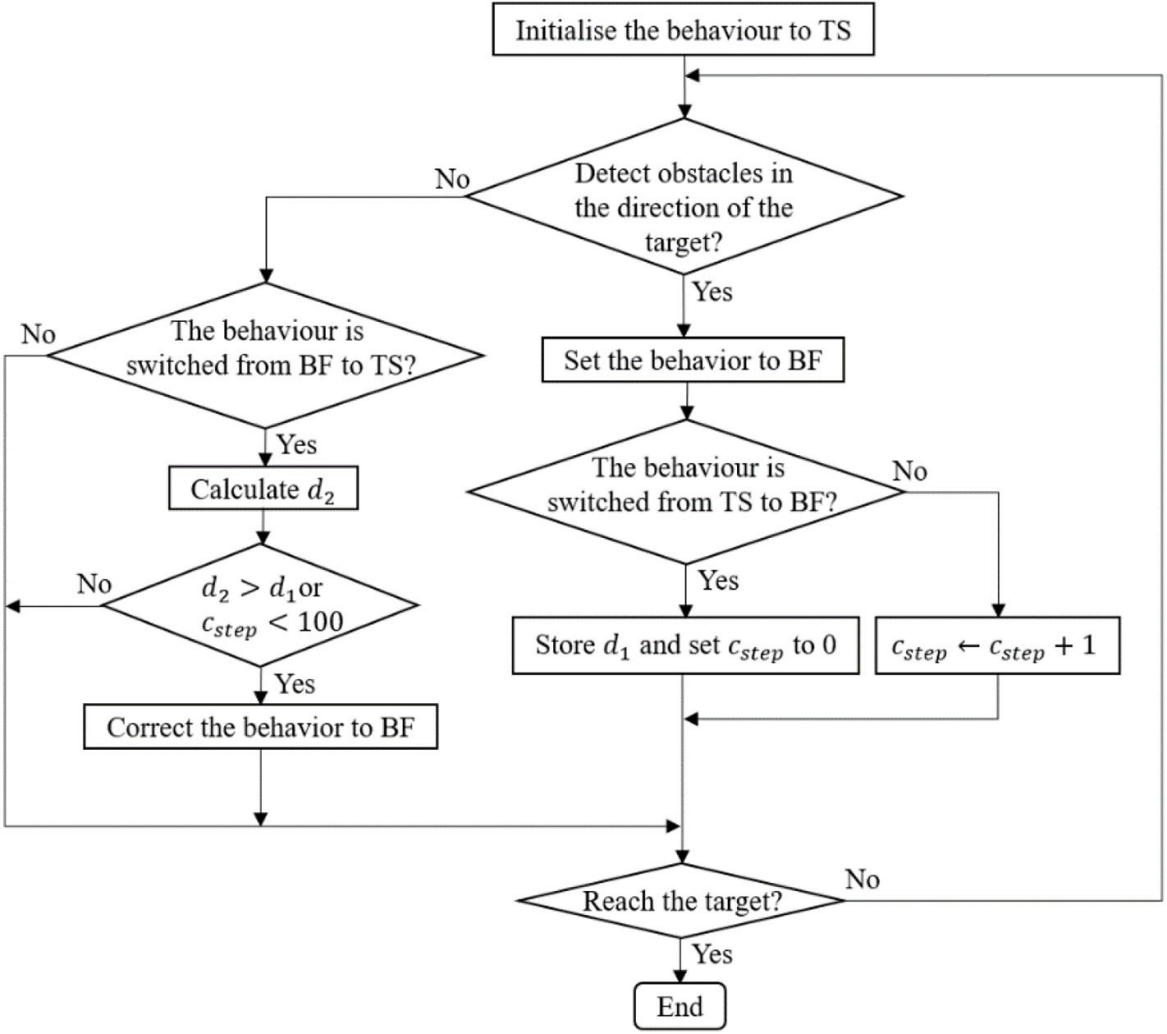
The block diagram of the navigation control.

In the simulation for robot navigation, the mobile robot is equipped with a 2D Lidar sensor that scans the area in front of the robot from right (0±) to left (180±). The coverage area is divided into eight sectors *L*_1_*,…,L*_8_, as shown in Figure 2. The behavior supervisor uses a simple logic proposed in Juang and Chang (2011) to switch between the target-searching (TS) behavior and left and right BF behavior. If there are no obstacles detected within the sensing range of the robot's Lidar, then the robot starts moving directly toward the target. Figure 5 shows the logic of the behavior selection based on the robot-target distance and time-step counter. When the robot switches behavior from target searching (TS) to BF, the distance *d*_1_ between the robot and the target is recorded, and the step counter *c*_*step*_ is set to zero. At the location where the robot decides to switch the behavior from BF to TS, the distance *d*_2_ between the robot and the target is calculated. If *d*_1_ > *d*_2_, or if the step counter *c*_*step*_ > 100, the robot keeps the original BF behavior; otherwise, the robot switches from BF to TS. This time-step constraint prevents the robot from immediately switching between the TS and BF behaviors.

The control of a robot performing BF in the navigation task is implemented by two fuzzy controllers—a left BF controller and a right BF controller. The left BF controller controls when the robot is closing an obstacle at the left-hand-side region, whereas the right BF controller is for the right-hand-side region. The number of rules in the right BF controller is identical to that in the left BF controller. The rules for the right BF behavior share the same antecedent part with those for the left BF behavior except that the left sensor inputs *L*_5_, …, *L*_8_ are changed to the right sensor inputs *L*_1_, …, *L*_4_. For the rule consequent part, the steering angle in each rule for the right BF behavior is simply a reverse of that for the left BF behavior. For example, suppose that the *i*th rule in the left BF controller is represented as follows:

(15) $$\begin{array}{l} R_{i}^{left}:\text{If }L_{5}\;is\;B_{i1}\text{ and }L_{6}\;is\;B_{i2}\;\ldots \text{ and }L_{8}\;is\;B_{i4}\text{ Then }\theta_{BF}\text{ is }\theta_{i}. \end{array}$$

Then, the corresponding rule for the right BF controller is

(16) $$\begin{array}{l} R_{i}^{right}:\text{If }L_{1}\;is\;B_{i1}\text{ and }L_{2}\;is\;B_{i2}\;\ldots \text{ and }L_{4}\;is\;B_{i4}\\ \text{Then }\theta_{BF}\text{ is }{-\theta }_{i}, \end{array}$$

where *B*_*i*1_, …, *B*_*i*4_ are a fuzzy sets defined by a Gaussian membership function and given as Equation (4). The output of the left BF controller is computed as Equation (6) with a singleton consequent value *a*_*i*_ = θ_*i*_; and similarly, the output of the right BF controller is with a consequent value *a*_*i*_ = −θ_*i*_. During a navigation task, the robot should decide to carry out either the left or right BF behavior at each control time step.

## Training Strategy and Simulation Configuration

In this study, both fuzzy-logic-based MRC and recurrent-based MRC are trained in a cascading manner. First, we train the BF controller in RBC to perform collision-free navigation toward the target. In the second phase of training, fuzzy-logic-based MRC and recurrent-based MRC learn to coordinate a group of RBC-equipped robots to arrive at a target at the same time. The particle swarm optimization (PSO) algorithm (Shi and Eberhart, 1998) is used to optimize the tunable parameters of all controllers.

### Particle Swarm Optimization

PSO is a swarm intelligence optimization approach in which each solution is represented as a particle [3]. Each particle has a position, represented by vector *s*_*i*_. The swarm in PSO is initialized with a population of random solutions. A swarm of particles moves through the solution space, and the velocity of each particle is represented by vector *v*_*i*_. The performance of a particle is measured by a fitness function *f*, which is evaluated using *s*_*i*_. Each particle keeps track of its own best position *p*_*i*_, which is associated with the best fitness that the particle has achieved. Also, it is guided toward the best position found by any member of the swarm (the global best position *g*). For particle *i* at iteration *t*, each element *k* of the new velocity can be calculated as

(17) $$\begin{array}{l} v_{i}^{\left(t\right)}\left(k\right)=wv_{i}^{\left(t-1\right)}\left(k\right)+c_{1}r_{1}\left(p_{i}\left(k\right)-s_{i}^{\left(t-1\right)}\left(k\right)\right)\\ \;+c_{2}r_{2}\left(g\left(k\right)-s_{i}^{\left(t-1\right)}\left(k\right)\right), \end{array}$$

where *w* is the inertia weight, *c*_1_ and *c*_2_ are positive acceleration coefficients, and *r*_1_ and *r*_2_ are uniformly distributed random numbers in the interval [0, 1]. All components of *v*_*i*_ have lower and upper bounds defined by the geometry of the search space. The new position of each particle is calculated with

(18) $$\begin{array}{l} s_{i}^{\left(t\right)}\left(k\right)=s_{i}^{\left(t-1\right)}\left(k\right)+v_{i}^{\left(t\right)}\left(k\right). \end{array}$$

With a careful choice of parameters *w, c*_1_, and *c*_2_, Equations (17) and (18) ensure that the particle population clusters around the best solution.

### Training Phase 1: BF Behavior

**Figure 6:**
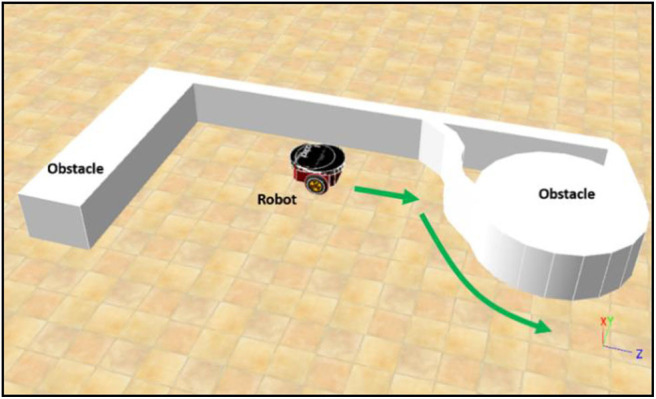
The environment for training phase 1.

Figure 6 illustrates the environment for training phase 1. The main goal of this phase is to control the robot in BF behavior at a constant speed using the PSO-based fuzzy controller. Without loss of generality, this value is set as 0.4 m/s in this paper. The BF behavior enables collision-free movement of the robot during navigation. Since only the left BF controller is trained, the distances detected by sectors *L*_5_, *L*_6_, *L*_7_, and *L*_8_ are used and fed as the inputs to the left BF controller. The right BF behavior is directly available by a slight modification of the learned consequents for the left BF behavior. The left BF controller output is the steering angle of the robot boundary-following behavior θ_*BF*_, where θ_*BF*_ ∈ [−3.14, 3.14] in radians. A positive value of θ_*BF*_ means a clockwise rotation.

The constraints for successful left BF behavior at each time step during the learning process are

(19) $$\begin{array}{l} \min\left(L_{5},L_{6},L_{7},L_{8}\right)>D_{min},\text{and }L_{5}\leq D_{max} \end{array}$$

In this simulation, *D*_*min*_ and *D*_*max*_ are set to 0.5 and 1.5, respectively. The first constraint prevents a collision with the object, and the second constraint prevents the robot from moving too far from the object. In the PSO-optimized training phase 1, a particle represents a whole fuzzy controller for left BF behavior. The performance of left BF behavior is evaluated as follows. The robot moves along the side of an object and stops when one of the constraints in (19) is violated, which indicates that the controller has failed. If the robot stops, the total number of control time steps is recorded as *T*_*control*_. The fitness function *f*_*phase*_1_ for training phase 1 is

(20) $$\begin{array}{l} f_{phase\text{\_}1}=\frac{1}{T_{control}}. \end{array}$$

A low *f*_*phase*_1_ indicates good left BF behavior. The control process from when movement starts to when it stops is called a trial. If left BF behavior fails, the robot moves back to its initial position for the next trial, and a new fuzzy controller is constructed and evaluated. The learning process is repeated until a successful fuzzy controller is found or the maximum number of iterations is met. A left BF behavior is deemed successful if it successfully controls the robot for a total of *T*_*suc*_ time steps. In the training phase 1, *T*_*suc*_ is set to 4,000 so that the robot moves along the object boundary for over two cycles. The maximum number of iterations is set to 200 for a trial.

### Training Phase 2: Multi-Robot Navigation and Arrival-Time Coordination Learning

**Figure 7:**
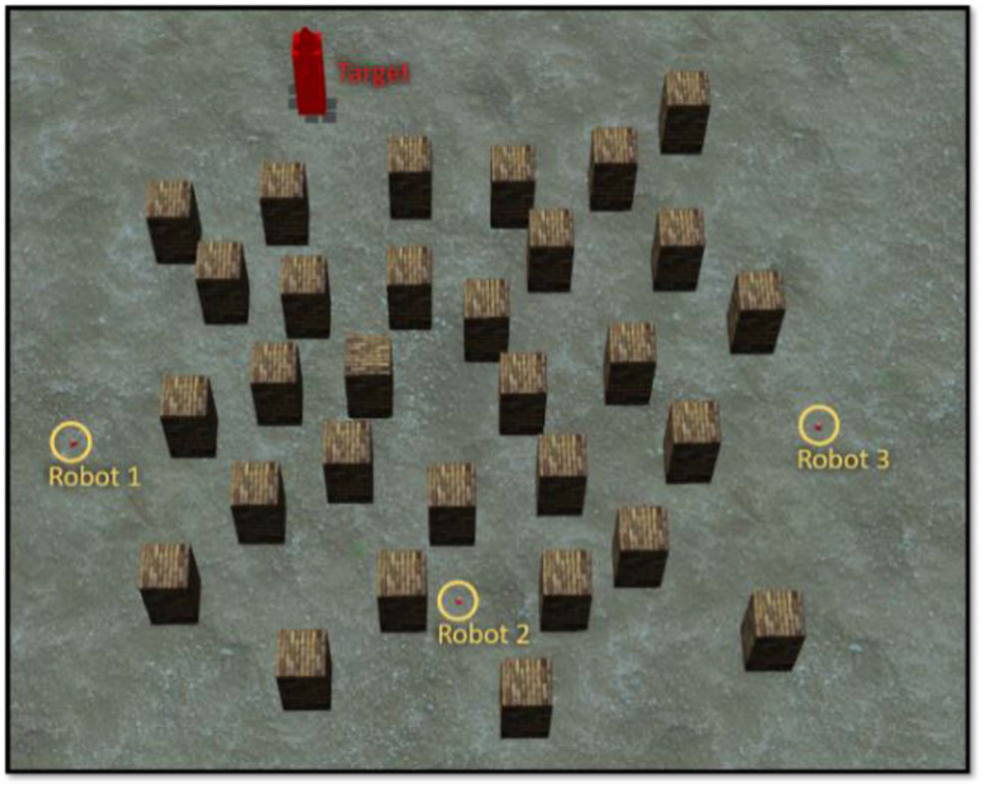
Environment for training phase 2.

In training phase 2, there are three robots moving in a complex environment, as shown in Figure 7. Each robot is controlled by the BRC whose BF controller was optimized in training phase 1. During training, both fuzzy-logic-based MRC and recurrent-based MRC are applied in the navigation of three robots so that they reach the target simultaneously. The robots start from different positions and head toward the same target. The performance of MRC is evaluated using a fitness function *f*_*phase*_ 2_:

(21) $$\begin{array}{l} f_{phase\text{\_}2}=10\cdot f_{1}+0.1\cdot f_{2}. \end{array}$$

The first term of (21), *f*_1_, is used to optimize the difference in the arrival times of *robot*_*rank*_1__ and *robot*_*rank*_*n*__:

(22) $$\begin{array}{l} f_{1}=|T_{rank_{1}}-T_{rank_{n}}|, \end{array}$$

where *T*_*rank*_1__ is the time that *robot*_*rank*_1__ takes to reach the target, and *T*_*rank*_*n*__ is the time for *robot*_*rank*_*n*__.

The second term of (21), *f*_2_, is to get the robot to move as fast as possible:

(23) $$\begin{array}{l} f_{2}=\frac{\left|T_{rank_{1}}-T_{rank_{n}}\right|}{2}. \end{array}$$

## Simulation Results

### Simulation 1 (BF Behavior Learning)

**Figure 8:**
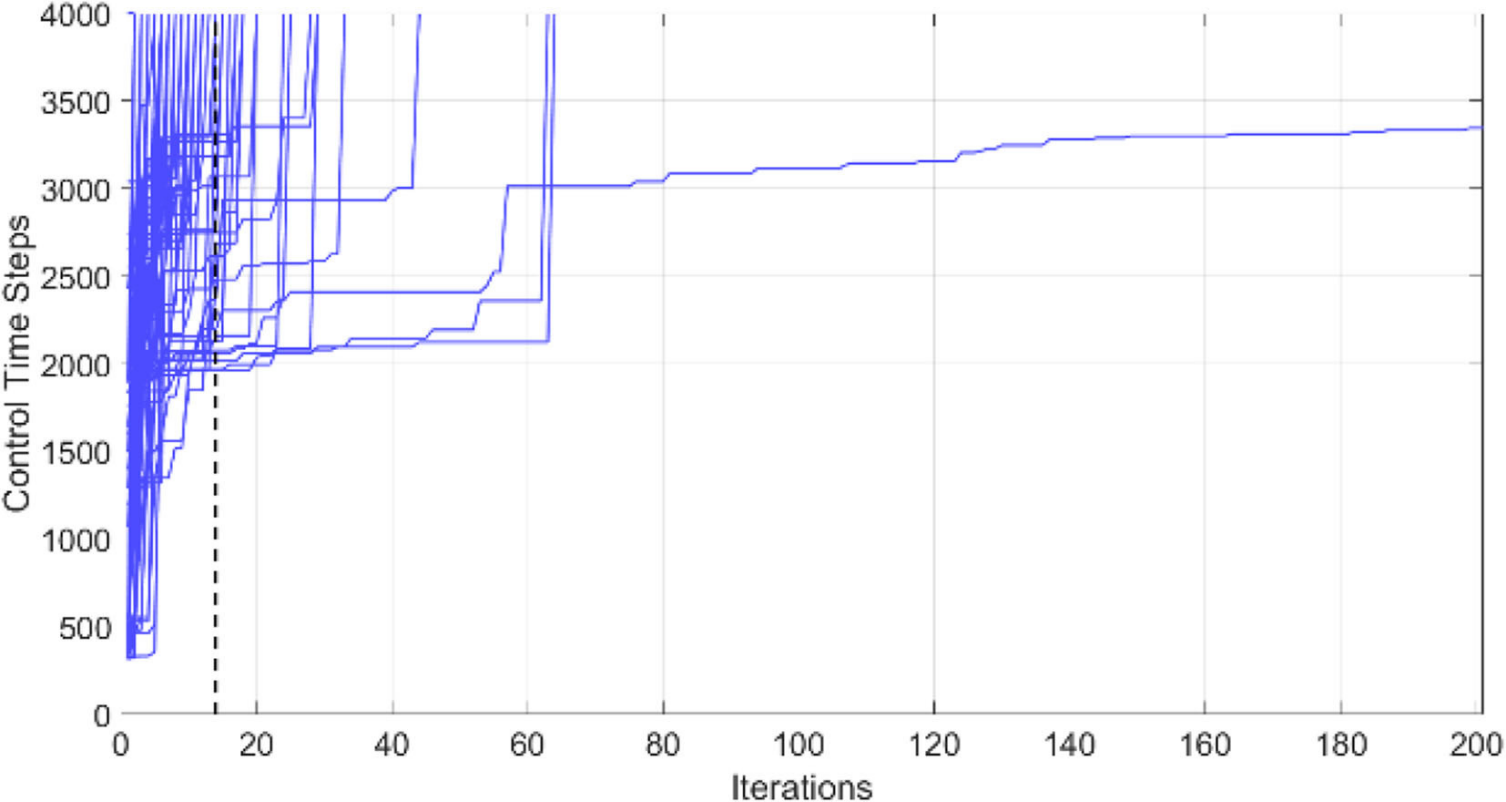
Robot left-BF behavior-learning results for all 50 runs.

This example shows the simulation results of training phase 1: left BF learning result using the PSO-optimized FLC. The number of fuzzy rules is set to 10. The simulation environment is as shown in Figure 6. The environment is built using Webots 8.5.3 on a platform equipped with Intel i5-4200H 3.40 GHz CPU, NVIDIA GT 745M 2GB graphics card, and 8G 1600 MHz RAM. The learning objective is to find a successful FLC for left BF behavior satisfying the constraints in (19) for a total of 4000 time steps. The control loop stops when the robot violates the constraint of the left-BF FLC. For this optimization problem, the objective is to design a successful FLC using as minimum number of iterations as possible. Figure 8 shows the left-BF behavior-learning results for all 50 runs. The PSO fails to find a successful left-BF FLC for one of the 50 runs. The average number of iterations of the PSO to find a successful FLC is 13.987.

### Simulation 2 (Multirobot Navigation and Arrival-Time Coordination Learning)

**Figure 9:**
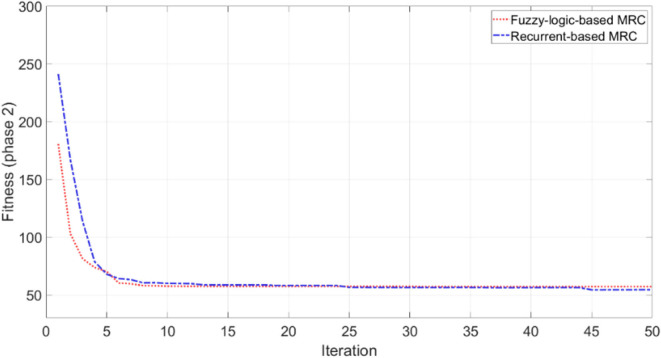
Average best-so-far fitness value at each iteration for the MRCs during the training phase 2.

The objective of this simulation is to optimize MRC for three robots to navigate and simultaneously reach the target in the clutter environment as shown in Figure 7. We set the centre point of the map as the origin of coordinate (x, z) = (0, 0), and the target is located at (x, z) = (−11, −23). The initial distance between *robot*_1_ is 30.4 m, *robot*_2_ is 39.5 m, and *robot*_3_ is 43.8 m. The performance of both fuzzy-logic-based MRC and recurrent-based MRC are evaluated by Equation (21). A smaller solution to Equation (21) means that the MRC can control the three robots moving toward to the target as fast as possible and coordinate their arrival time as precise as well. For each evaluation process, the MRC controls all robots until they all reach the target. Once all robots have reached the target, the positions of the robots will be set to their initial positions, which are fixed during the whole training process. The number of learning iterations is set to 50. The PSO optimization process for training phase 2 conducts 50 runs for statistical evaluation. Figure 9 demonstrates the average best-so-far fitness of the MRCs. Table 1 presents the performance of fuzzy-logic-based MRC and recurrent-based MRC in training phase 2. The average best-so-far fitness of fuzzy-logic-based MRC converges at 57.2 (average value of *f*_1_ is 532.0 time steps, average value of *f*_2_ is 0.41 time steps), while the recurrent-based is 54.46 (average value of *f*_1_ is 524.62 time steps, average value of *f*_2_ is 0.20 time steps).

**Table 1:** Performance of fuzzy-logic-based and recurrent-based MRC in the training phase 2.

		***f*_*phase*_2_**	***f*_1_**	***f*_2_**
Fuzzy-logic-based MRC	Average	57.73	532.0	0.41
	STD	2.97	29.85	0.068
Recurrent-based MRC	Average	54.46	524.62	0.20
	STD	3.31	32.42	0.053

### Simulation 3 (Multirobot Navigation and Arrival-Time Coordination)

In this simulation, the optimized MRCs and BF controller are applied to perform navigation and arrival-time task. We deployed three robots and six robots in a clutter environment with variant starting position to testify the scalability of the optimized MRCs. Both fuzzy-logic-based and recurrent-based MRCs are used in this simulation. To demonstrate the ability of time-arrival coordination, examples of robots controlled without MRC are included for comparison with those examples applied with MRCs.

#### Three-Robot Navigation and Arrival Time Control

**Figure 10:**
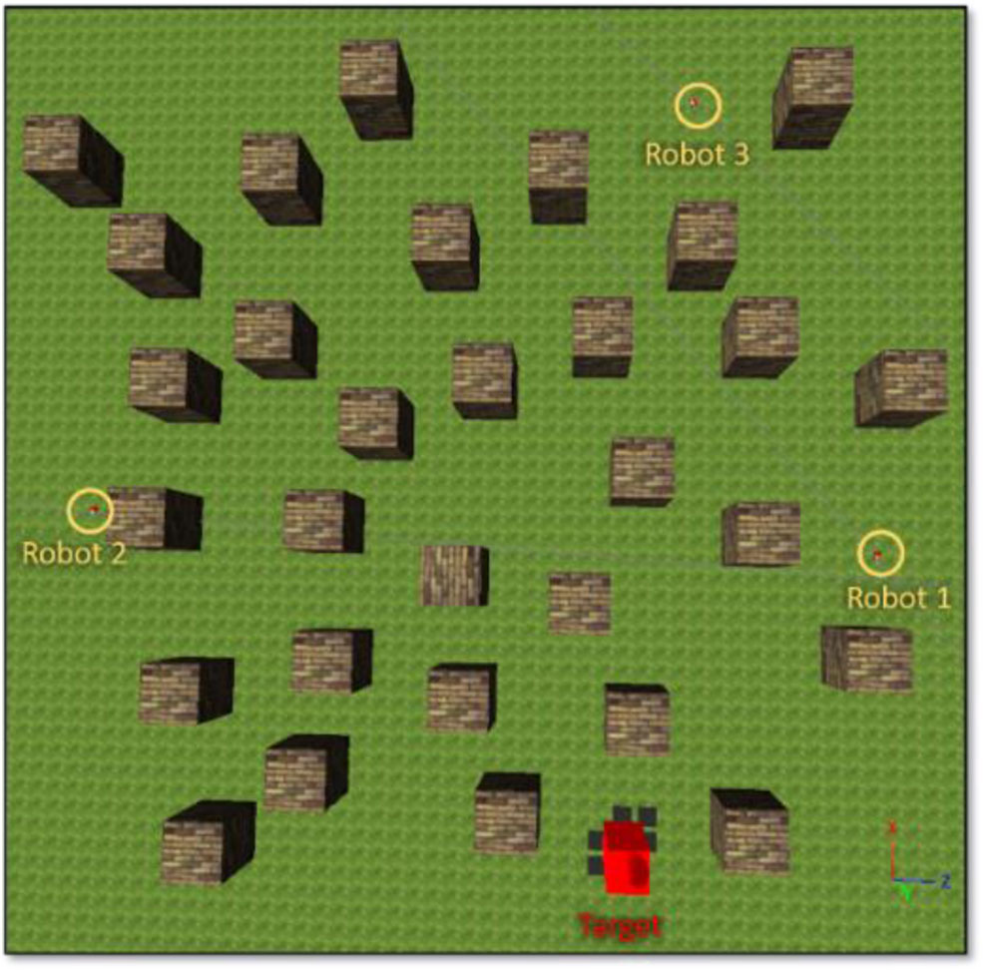
An environment setting for three-robot navigation.

**Figure 11:**
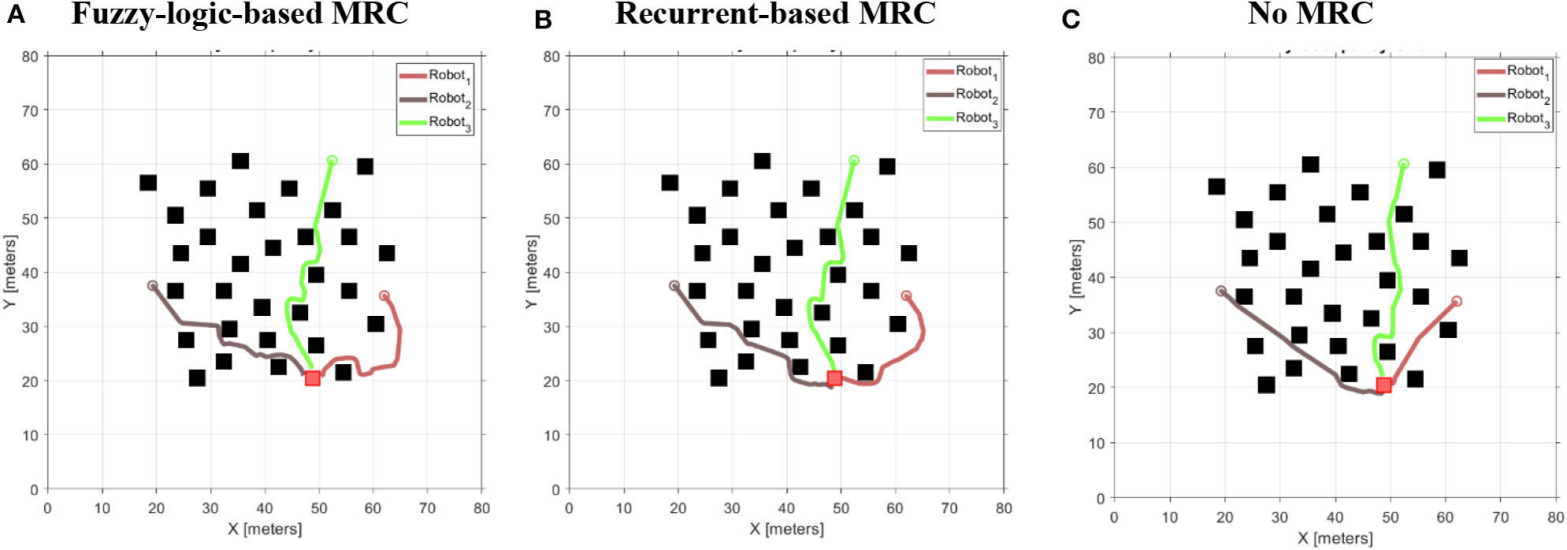
The moving speeds of four-robot navigation in a complex environment setting. **(A)** Robots are controlled by the fuzzy-logic-based MRC. **(B)** Robots are controlled by the recurrent-based MRC. **(C)** Robots are controlled by only their own RBC.

**Figure 12:**
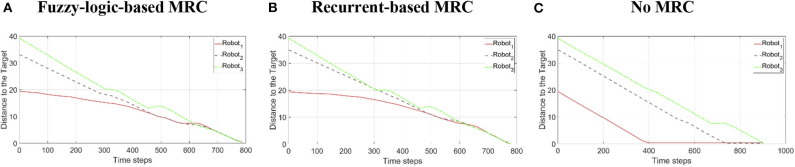
The remaining distance between the target and the three robots during the three-robot navigation and arrival time control simulation. **(A)** Robots are controlled by the fuzzy-logic-based MRC. **(B)** Robots are controlled by the recurrent-based MRC. **(C)** Robots are controlled by only their own RBC.

To validate the performance of the proposed control systems, we deployed three robots in a complex environment (see Figure 10), and the target building is set at (*x, z*) = (−19.54, 8.78). The initial distance between the *robot*_1_ is 19.34 m, *robot*_2_ is 32.94 m, and *robot*_3_ is 39.05 m. Figure 11 illustrates the trajectories of the three robots, respectively, controlled by fuzzy-logic-based MRC, recurrent-based MRC, and in the absence of MRC during the navigation task. Figure 12 shows the remaining distance between the target and the three robots. The performance of time arrival coordination with three-robot setting is shown in Table 2. The time difference is evaluated by measuring the difference of arrival time of the fastest robot and the slowest robot in the simulation. The best achieved time difference controlled by fuzzy-logic-based MRC is five time steps, while it takes 785 time steps for the slowest robot to complete the navigation task. The recurrent-based configuration showed a result with an eight-time-step difference between the first and last arriving robots, while it takes 778 time steps for the slowest robot to complete the navigation task. By comparing Figures 12A,B, the robots controlled by recurrent-based MRC have faster convergence speed, but fuzzy-logic-based has a better coordinating ability in this simulation. We further compare with the case without MRC control; see Figures 11C, 12C. The three robots directly move toward to the target without changing their searching direction and the moving speed so that *robot*_1_ arrives at the target much earlier than the other two robots.

**Table 2:** The performance of time arrival coordination with three-robot setting.

**Unit: Time step**	**Time difference**	**Time to complete task**
Fuzzy-logic-based MRC	5	785
Recurrent-based MRC	8	778
No MRC	504	901

#### Six-Robot Navigation and Arrival Time Control

**Figure 13:**
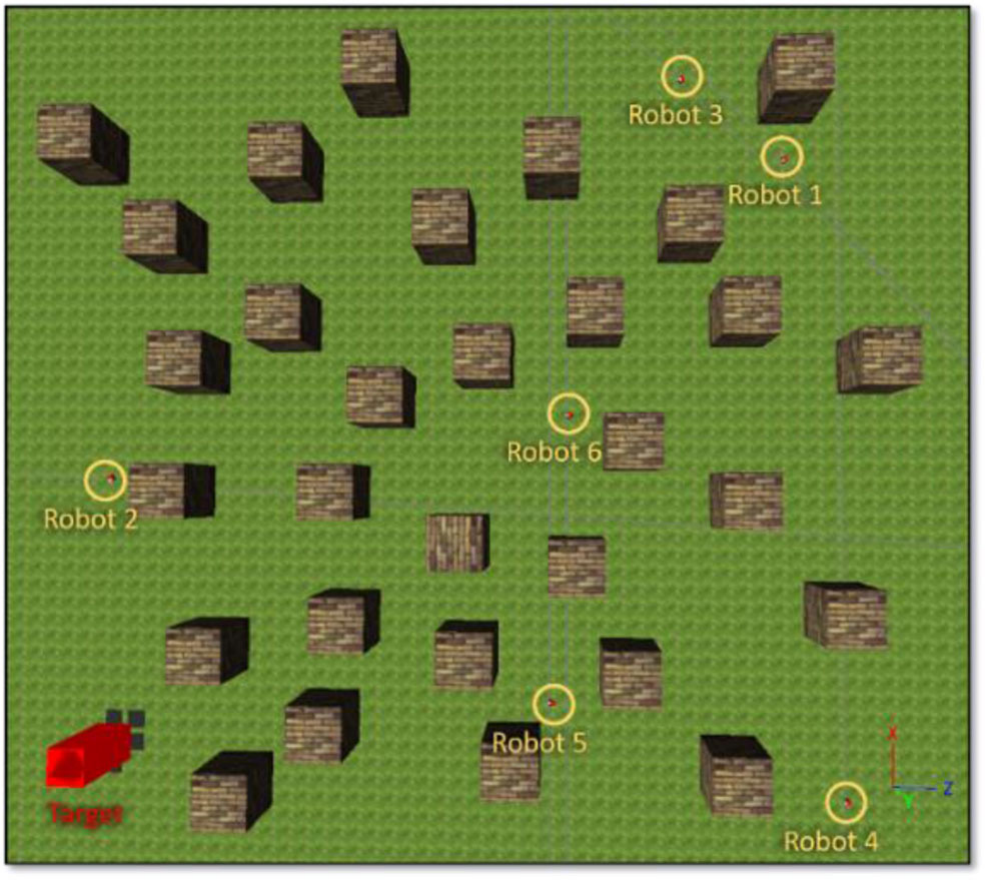
An environment setting for six-robot navigation.

The reason for us to use the simple interpolation method to decide the velocity scaling factors and searching direction of robots is to increase the scalability of the proposed methods such that they can deal with a different and changing number of robots without retraining the neural networks. To validate the scalability of the proposed methods, we deployed six robots in this simulation, as shown in Figure 13, and the target building is set at (*x, z*) = (−17.71, −20.34). The initial distance between *robot*_1_ is 50.11 m, *robot*_2_ is 15.26 m, *robot*_3_ is 49.44 m, *robot*_4_ is 41.69 m, *robot*_5_ is 24.08 m, and *robot*_6_ is 34.27 m. Each proposed model uses identical parameters, which are used in the simulation of section IV-C.

**Figure 14:**
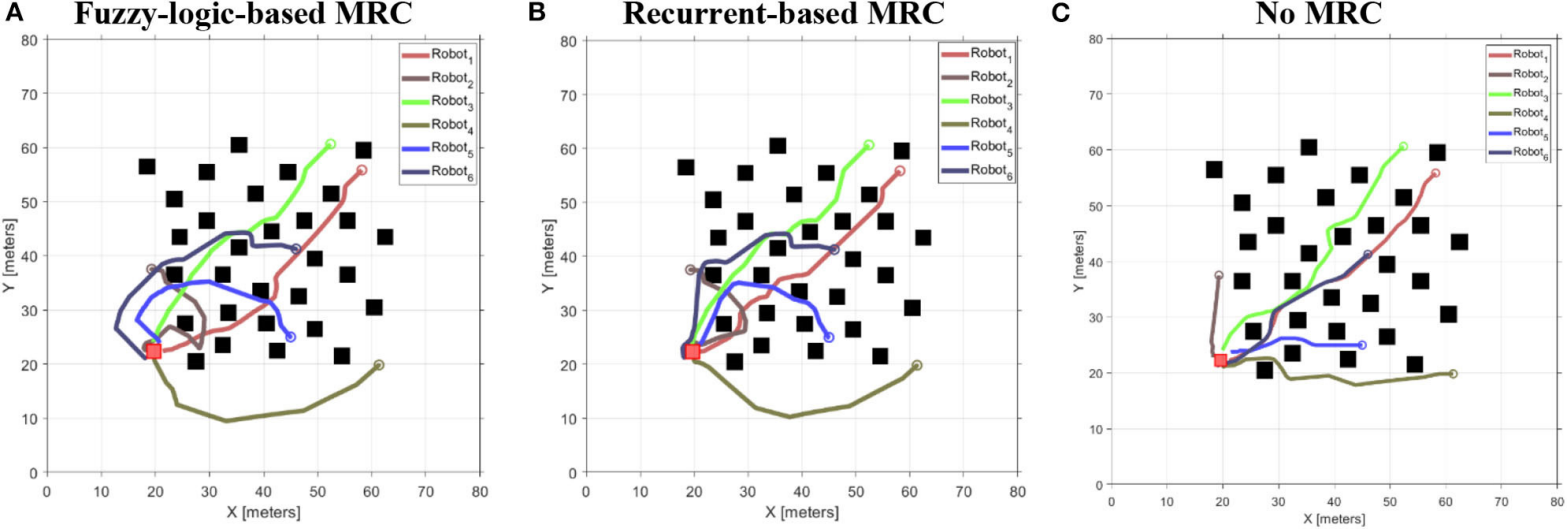
The remaining distance between the target and the three robots during the three-robot navigation and arrival time control simulation. **(A)** Robots are controlled by the fuzzy-logic-based MRC. **(B)** Robots are controlled by the recurrent-based MRC. **(C)** Robots are controlled by only their own RBC.

**Figure 15:**
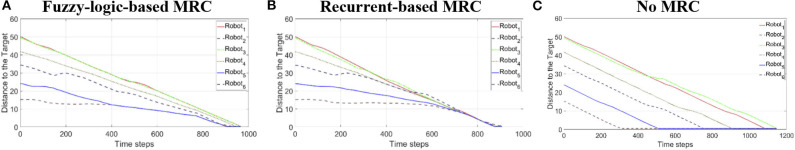
The moving speeds of six-robot navigation in complex environment setting. **(A)** Robots are controlled by the speed coordination controller. **(B)** Robots are controlled by the improved controller. **(C)** Robots are controlled by only their own RBC.

The trajectories of the six-robot setting controlled by each proposed model are illustrated in Figure 14. Figure 15 shows the remaining distance between the target and the six robots. Table 3 presents performance of the arrival-time coordination in the six-robot setting. Fuzzy-logic-based MRC achieves 68-time-step time difference between the fastest robot and the slowest robot, while it takes 965 time steps for the slowest robot to complete the navigation task. The recurrent-based MRC has a better result with a 34-time-step difference between the first and last arriving robots, while it takes 907 time steps for the slowest robot to complete the navigation task. The robots controlled by recurrent-based MRC have faster convergence speed, as well as smaller time difference between the fastest robot and the slowest robot. In this simulation, recurrent-based MRC shows its better performance in arrival-time control than fuzzy-logic-based MRC does. The control results also demonstrated that the proposed models have the scalability and are both able to navigate different number of robots without retraining the model.

**Table 3:** The performance of time arrival coordination with six-robot setting.

**Unit: Time step**	**Time difference**	**Time to complete task**
Fuzzy-logic-based MRC	58	965
Recurrent-based MRC	34	907
No MRC	839	901

## Conclusion and Future Work

We developed a fuzzy-logic-based coordinator and a recurrent-based coordinator for safely navigating multiple robots in cluttered environments, where the controller regulates their speeds and adjusts their searching direction to enable simultaneous arrival time on targets. The environment for the test was designed in an imbalanced setting, in which each robot starts at the positions with totally different distances to the target. The simulation results demonstrate that the two proposed models successfully control a different number of robots to safely navigate and reach the target on time. In the future, we intend to develop a systematic method for optimizing the hyper-parameters of this cascaded model, including the number of rules in FLS. We are currently designing a technique for automatically tidying up the membership functions for improved interpretability. We will also consider scenarios where the robots lose communication when they are separated by building structures, losing a direct line of sight. We believe that intermittent sharing of controller policy will allow the agent to predict the future motions of the other agents when the communication is blocked and to make correct motion decisions.

## Data Availability Statement

The datasets generated for this study are available on request to the corresponding author.

## Author's Note

We developed a fuzzy-logic-based coordinator and recurrent-based coordinator for safely navigating multiple robots in clutter environments, where the controller regulates their speeds and adjusts their searching direction to enable simultaneous arrival time on targets. The environment for the test was designed in an imbalanced setting, in which each robot starts at the positions with totally different distances between it and the targets. The simulation results demonstrate that the two proposed models successfully control a different number of robots to safely navigate and reach the target on time.

## Author Contributions

Y-CC developed the methodology, performed the experiments, and wrote the manuscript. AD proposed the experiments and partially contributed to the manuscript. The project was administered by C-TL and JK.

## Conflict of Interest

The authors declare that the research was conducted in the absence of any commercial or financial relationships that could be construed as a potential conflict of interest.
